# Efficacy of abdominal trunk muscles-strengthening exercise using an innovative device in treating chronic low back pain: a controlled clinical trial

**DOI:** 10.1038/s41598-020-78908-9

**Published:** 2020-12-14

**Authors:** Ryo Kitagawa, Satoshi Kato, Satoru Demura, Yuki Kurokawa, Kazuya Shinmura, Noriaki Yokogawa, Noritaka Yonezawa, Takaki Shimizu, Norihiro Oku, Makoto Handa, Ryohei Annen, Hiroyuki Tsuchiya

**Affiliations:** Department of Orthopaedic Surgery, Graduate School of Medical Sciences, Kanazawa University, 13-1 Takara-machi, Kanazawa, 920-8641 Japan

**Keywords:** Rehabilitation, Pain management

## Abstract

Exercise is the most common conservative intervention for chronic low back pain (CLBP). We have developed an innovative exercise device for the abdominal trunk muscles that also measures muscle strength in a sitting position. The device, which is easy for patients with CLBP to use, allows for lumbar stabilization exercise under pressure. This study aimed to examine the efficacy of abdominal trunk muscle strengthening using the device in improving CLBP. We conducted a two-group non-randomized controlled clinical trial. CLBP patients were allocated into two groups. The strengthening group underwent a 12-week exercise program that included abdominal trunk muscle strengthening using our device and stretching exercises, while the control group received a 12-week stretching exercise program. The outcome measures included the improvement of the abdominal trunk muscle strength measured by the device, pain intensity of CLBP, physical function, and quality of life (QOL). A total of 40 participants (20 in each group) were analyzed. The strengthening group showed better improvement in the abdominal trunk muscle strength, CLBP, physical function, and QOL than in the control group. In conclusion, the strengthening exercise using the device with easy stretching was effective in improving the strength of the abdominal trunk muscles, pain intensity of CLBP, physical function, and QOL.

## Introduction

Low back pain (LBP) is one of the common health problems worldwide^1^. It affects approximately 80% of people at some stage in their lifetimes^2,3^, and remains a major cause of disability and morbidity^4–6^. LBP is also associated with high financial costs^7^. In addition to medical treatment, musculoskeletal physiotherapy (including exercise and manual therapy) is one of the most common non-invasive and conservative interventions for LBP^8–10^.

Supervised exercise therapy was recommended as the first-line treatment for the management of chronic non-specific LBP in several guidelines^1,11–13^. However, systematic reviews arguing for exercise therapies have not concluded which form of exercise was superior to others^14,15^.

Stabilization exercise programs have been widely applied for LBP treatment because they were effective in reducing LBP and disability^14,16^. Stabilization exercises aiming to improve the function of specific trunk muscles supposedly control the movement of the spine. Through the regaining control and coordination of the spine and pelvis, these exercises could be effective in the treatment of LBP^17,18^.

**Figure 1:**
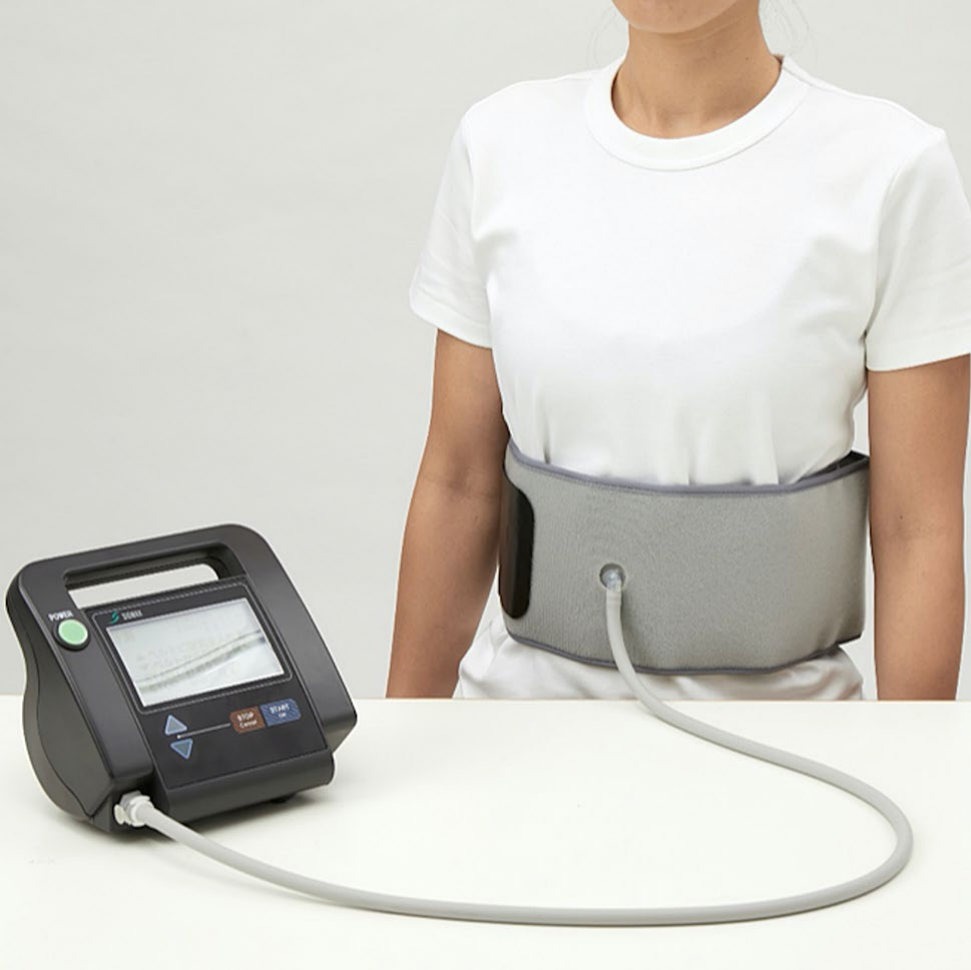
A new exercise device for the abdominal trunk muscles. This image shows an inflatable cuff around the abdomen and a mechanical manometer to measure pressure. The device enables the strengthening of abdominal trunk muscles and strength measurement in a sitting position without causing lower back pain (the image is the authors' work).

An innovative exercise device has been developed for the abdominal trunk muscles (RECORE; Nippon Sigmax Co., Ltd., Shinjuku-ku, Tokyo, Japan; Fig. 1)^19^. The device enables strengthening of abdominal trunk muscles and strength measurement in a sitting position without requiring painful lower back movements. Moreover, the device has a built-in system for measuring abdominal trunk muscle strength, which may reinforce adherence to the exercise program. In addition, the exercise using the device is classified as “lumbar stabilization exercise under pressure”. A previous study showed that strengthening exercise with the device increased muscle strength and activated the abdominals, diaphragm, and pelvic floor muscles, which in turn could stabilize the spine^20^. A pilot study also demonstrated that the exercise using the device is safe even among elderly individuals^21^.

This study aimed to examine the efficacy of abdominal trunk muscle strengthening using the device in improving the pain intensity of chronic low back pain (CLBP), physical function, and quality of life (QOL) among patients with CLBP.

## Methods

We conducted this two-group non-randomized controlled clinical trial between March 2018 and March 2019 in the Department of Orthopaedic Surgery at the university hospital. All participants were diagnosed with CLBP by a physician and were referred to our institute. The inclusion criteria were as follows: diagnosis of CLBP (at least 3 months) by a physician, age > 40 years, LBP of at least 3 on an 11-point numerical rating scale (0, no pain; 10, worst pain) at study registration, and the capability of performing the prescribed exercise regimen. On the contrary, the exclusion criteria included considerable neurological signs or specific spinal pathology (e.g., malignancy, infection, or acute vertebral fracture); a history of spinal surgery; severe osteoporotic spine; severe medical comorbidities (e.g., cardiovascular, respiratory, or renal disease); comorbid rheumatologic disease; and comorbid dementia. After providing an explanation of the trial, written informed consent was obtained from all patients before participation. This study conformed to the Declaration of Helsinki and was approved by the Ethics Committee of Kanazawa University Hospital. This study was also prospectively registered with the UMIN Clinical Trial Registry (UMIN 000031168; 06/02/2018). All institutional and national guidelines for the care and use of participants were followed.

Forty-four participants were enrolled and performed the prescribed exercise regimen. All patients were clinically examined with magnetic resonance imaging to determine if they met the aforementioned criteria. The participants were divided into two groups as follows: the strengthening group and the control group. Twenty-three patients enrolled in the trial in odd-numbered months (January, March, May, July, September, and November) and were allocated to the strengthening group, whilst 21 patients enrolled in even-numbered months (February, April, June, August, October, and December) and were assigned to the control group.

### Exercise device

#### Measurement mode

**Figure 2:**
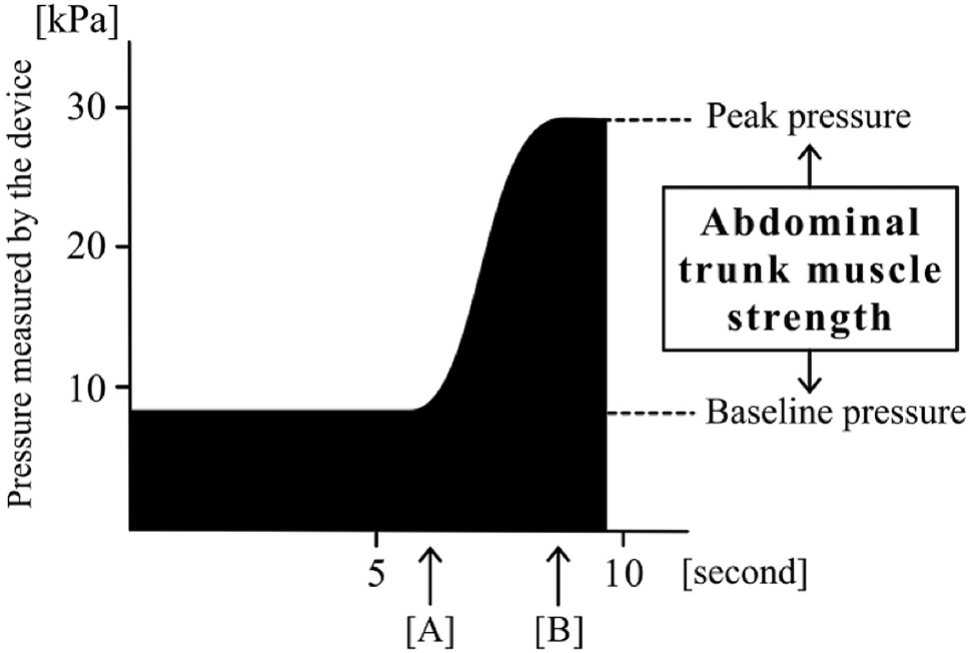
This waveform shows the time course of the pressure measured by the cuff during measurement mode. (**a**) The point at which the subject started to contract the abdominal trunk muscles against the pressure. (**b**) After the pressure reached a peak, it automatically decreased as the air in the cuff was released.

As described in detail previously^19,20^, the device’s design is similar to that of a sphygmomanometer. It has an inflatable cuff and a mechanical manometer to measure pressure. For measurement, the subject puts the cuff around the abdomen in a sitting position. Thereafter, the subject inflates it to apply adequate pressure to the abdominal wall (i.e., the baseline pressure; Fig. 2). Under the baseline pressure, the subject exerts maximum force by contracting the abdominal trunk muscles. Consequently, the pressure in the cuff is elevated and reaches a peak (i.e., the peak pressure; Fig. 2). The mechanical manometer calculates and reports a pressure value, which is a result of subtracting the baseline pressure from the peak pressure and represents the muscle strength value (measured in kPa) in a numerical form. After the pressure reaches a peak, it automatically decreases as the air in the cuff is released. The muscle strength value was considered as the abdominal trunk muscle strength in this study.

A validation study demonstrated that the device could measure the strength of the abdominal trunk muscles and had an excellent intra-rater and inter-rater reliabilities for the measurement of muscle strength^20^.

#### Training mode

**Figure 3:**
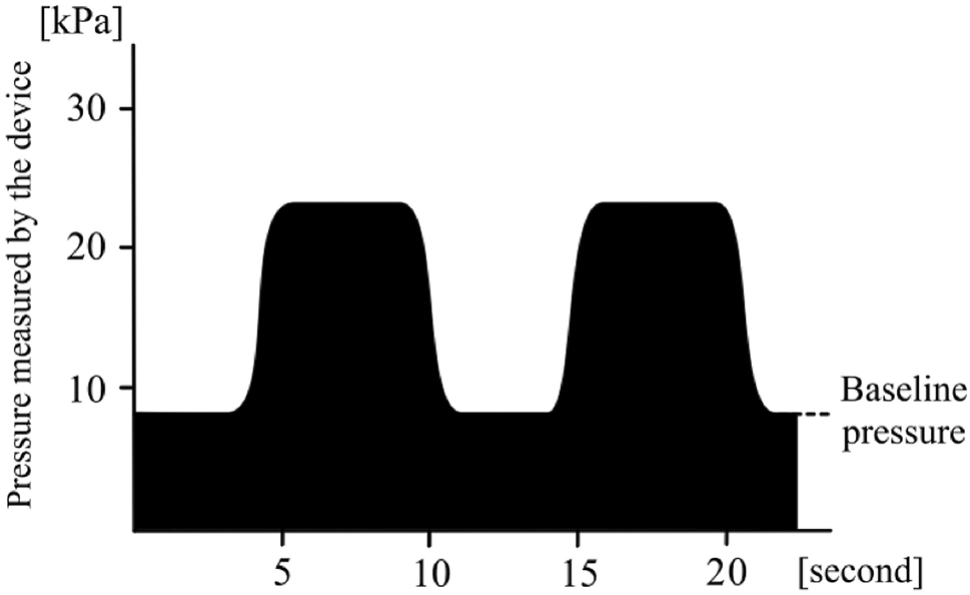
This waveform shows the time course of the pressure measured by the mechanical manometer in the device during the training mode. The exercise using the device allows the subjects to easily and powerfully contract the abdominal trunk muscles under the baseline pressure. During the exercise, the device displays the abdominal muscle strength in real-time as a waveform and a numerical form.

During the muscle strengthening exercise using the device, the subject intermittently or continuously contracts the abdominal trunk muscles under pressure from the cuff. This exercise is similar to a bracing exercise and functions as a stabilization exercise^22^. The exercise using the device allows the subjects to easily and powerfully contract the abdominal trunk muscles. During the exercise, the device displays the abdominal trunk muscle strength in real-time as a waveform and a numerical form (Fig. 3).

#### Run-in period (preparation)

A run-in period of 2 weeks was applied to ensure the stability of the participants with CLBP before commencing the intervention. During the run-in period and the trial period, all participants were requested to discontinue pain relievers (excluding loxoprofen sodium), other exercises, and local injection. Of the 16 patients who were administered pain relievers at the registration of the trial, administration of pain relievers was discontinued in six patients during the run-in period; 10 patients continued loxoprofen sodium during the run-in and trial periods.

### Intervention

#### Strengthening group

**Figure 4:**
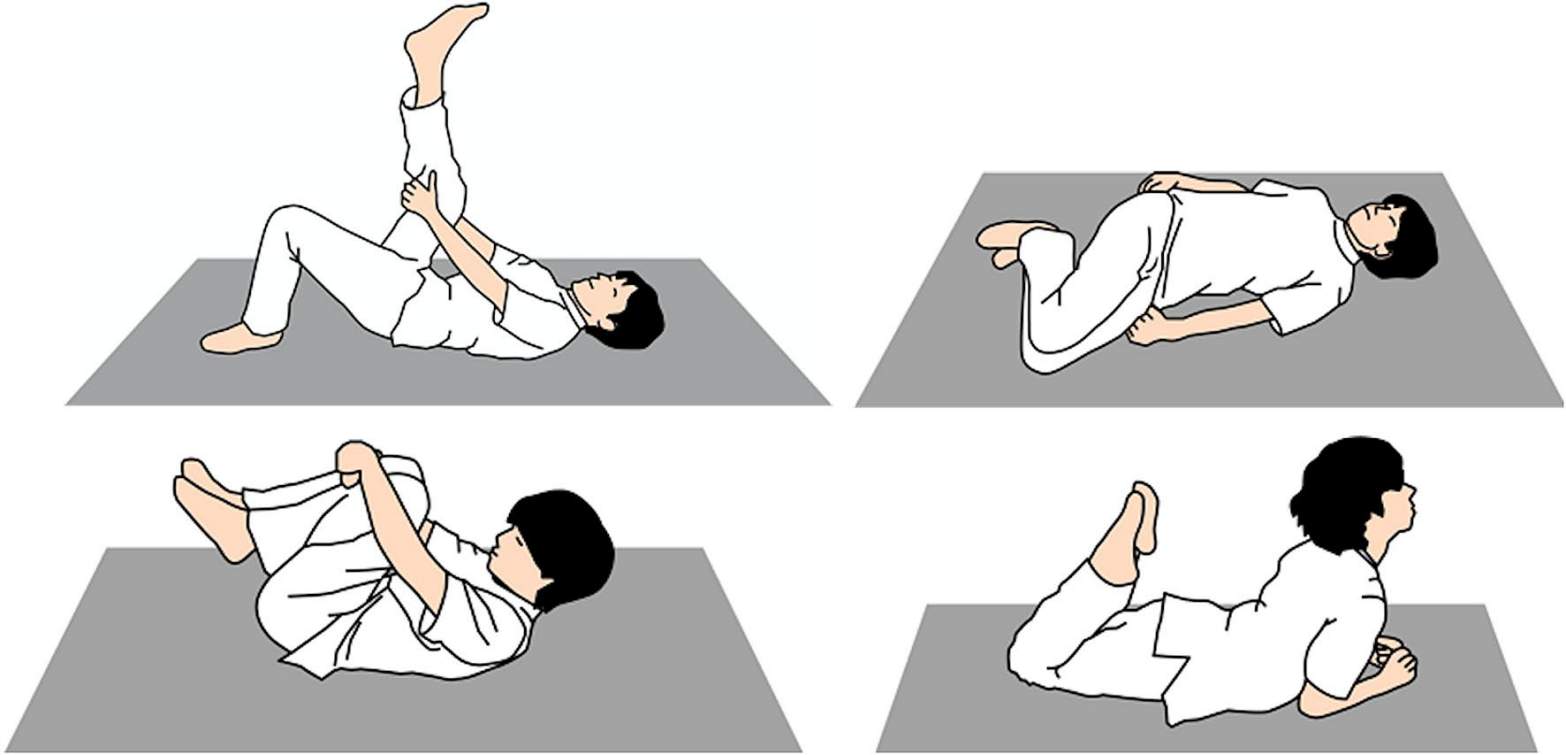
Four types of stretching were performed to stretch the abdominal and back muscles, iliopsoas, gluteal muscles, and hamstrings and to mobilize the lumbar spine.

Participants in the strengthening group underwent a 12-week exercise program, which included abdominal trunk muscle strengthening and stretching exercises. The strengthening exercise consisted of two 10-min sessions of abdominal trunk muscle strengthening and was performed using the device. During the strengthening exercise, the participants intermittently contracted their abdominal muscles under the pressure applied by the cuff. They exerted the force necessary for the cuff pressure to reach 50–80% of the peak pressure measured at the beginning of the exercise as the repetition of light to moderate exercise is recommended for older adults to increase power^23^. The intermittent muscle contraction was performed once every 10 s, with 5 s of muscle contraction and 5 s of rest. In the stretching exercise, four types of stretching were performed (Fig. 4), which aimed to stretch the abdominal and back muscles, iliopsoas, gluteal muscles, and hamstrings and to mobilize the lumbar spine. The participants performed both the strengthening and stretching exercises in the rehabilitation room in our hospital. All sessions were facilitated by a researcher, and the whole exercise program was completed within approximately 30 min. The participants were asked to visit our hospital three times per week for 12 weeks; thus, a total of 36 exercise sessions were conducted in the trial. Adverse events, abnormal changes in vital signs, and pain exacerbation were not observed during and after the exercise using the device in our pilot study^21^. A home stretching exercise program was also provided to the participants, along with diaries to record the number of exercises they performed.

#### Control group

Participants in the control group underwent a 12-week stretching exercise program similar to that in the strengthening group. In addition, they were asked to visit our hospital once a week for 12 weeks; thus, a total of 12 exercise sessions were conducted in the trial. Participants equally received a home stretching exercise program and diaries.

### Primary outcomes

Primary outcomes were measured at baseline (on entry to the trial), 4 and 8 weeks (during intervention), 12 weeks (at the end of the intervention), and 24 weeks (12 weeks after the intervention).

#### Trunk muscle strength

The novel exercise device was used to measure the abdominal trunk muscle strength (kPa). The subjects performed two trials, and the trial with the better result was used in the analysis.

#### Pain

LBP was assessed using patient-reported numerical rating scale (NRS; range 0 [no pain] to 10 [worst pain]). The patients were asked to rate their LBP during the last few days. NRS has excellent reliability^24^, and the minimally clinically important difference (MCID) of LBP was 2 points^25^.

#### Disability

The Roland Morris Disability Questionnaire (RDQ) was used to measure limitation in activity^26^. This disability questionnaire has 23 items (0–23 scale). RDQ has excellent reliability^27^, and the MCID was 4 points^28^.

### Secondary outcomes

Secondary outcomes were measured at baseline and 12 weeks (the end of intervention).

#### Physical function

Finger-floor distance (FFD; cm) was evaluated to determine trunk flexibility. Timed up and go test (TUG) was used for measuring the time needed by the participants to stand up from the chair, walk 3 m to a red marker, and return to the starting position on the chair. The TUG is a useful test to evaluate basic mobility skills^29^. The one-leg standing (OLS) test was performed to assess physical balance and to measure the time one could stand on one lower limb with eyes open^30^. FFD, TUG, and OLS tests had excellent reliability^31–33^, and the MCIDs were 4.5 cm, 3.4 s, and 24.1 s, respectively^33–35^.

#### General health

QOL was assessed by the Medical Outcomes Study Short-Form 36-Item Health Survey (SF-36), a standardized instrument that measures health status-related QOL. SF-36 has eight domains: physical functioning, role physical, bodily pain, general health, vitality, social functioning, role emotional, and mental health. The scores range from 0 to 100, with higher scores denoting better QOL^36,37^. SF-36 had good reliability^37^, and the MCIDs is 5 points^38^.

#### Adherence

Participants recorded the number of days the exercise sessions were performed, including home stretching exercises, in their diaries.

### Sample size

Based on the data from a previous study^21^, 42 participants were needed to detect a between-group difference in LBP improvement, with a power of 0.80, a two-sided alpha of 0.05, and 10% attrition.

### Statistical analysis

Data were presented as median and interquartile range. Friedman test was used to evaluate the effects of the interventions and the differences in the primary outcomes (i.e., abdominal trunk muscle strength, NRS of LBP, and RDQ) at 0 (baseline), 4, 8, 12, and 24 weeks in both groups. The Scheffe test was conducted as a post-hoc test. The Wilcoxon signed-rank test was used in the evaluation of the differences in the secondary outcomes at 0 (baseline) and 12 weeks in both groups. The Mann–Whitney U test was employed to determine the difference in outcome improvement between the two groups. Values of *P* ˂ 0.05 were considered statistically significant. Statistical analyses were performed using the Statistical Package of Social Sciences software version 25.0 for Mac (SPSS Inc., Chicago, Illinois, USA).

## Results

### Demographics

**Figure 5:**
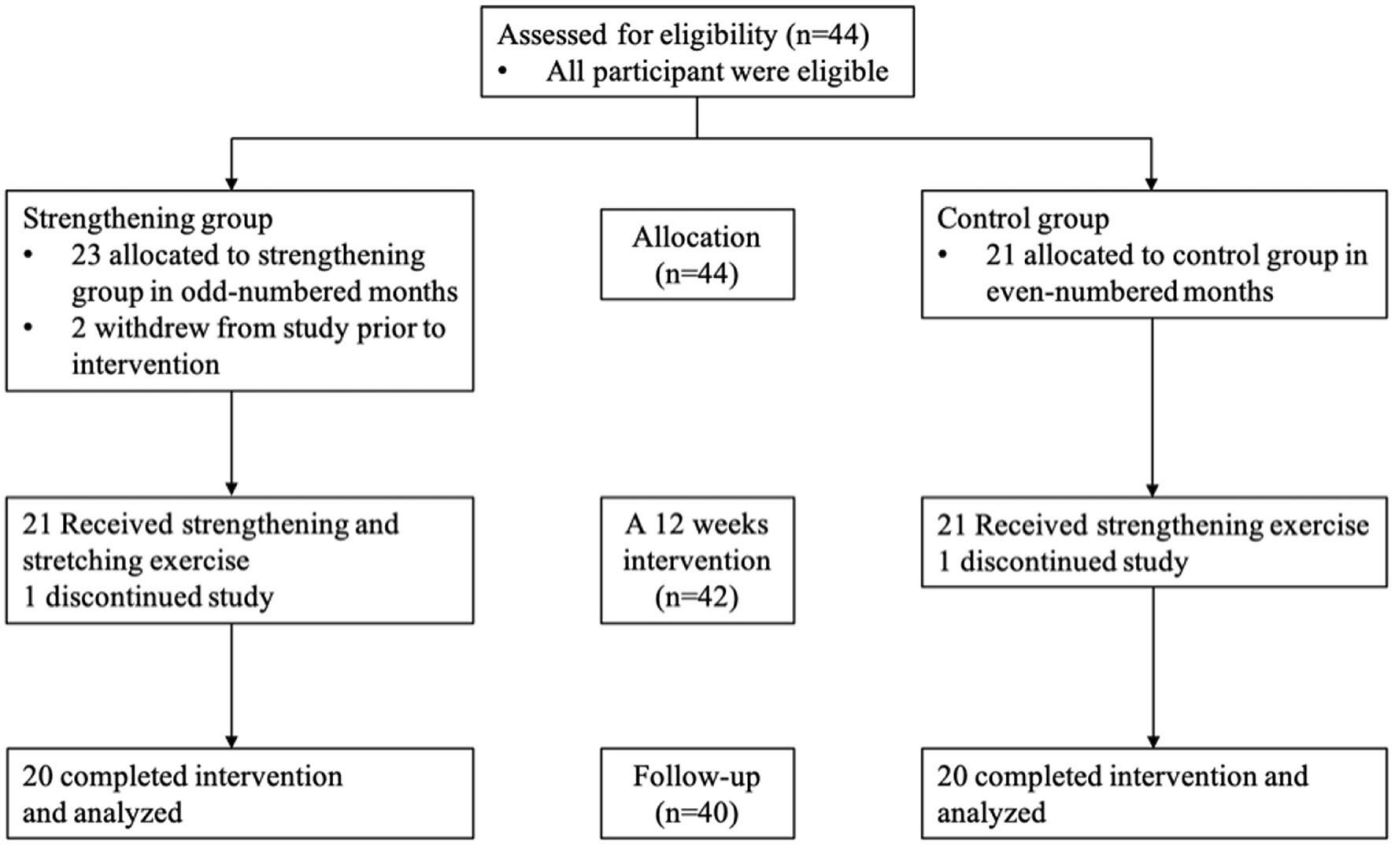
Flow of the participants throughout the trial. The participants were divided into two groups as follows: the strengthening group and the control group. A total of 40 participants (20 in each group) were analyzed.

All of the 44 participants enrolled between March 2018 and March 2019 were eligible to participate. Twenty-three were allocated to the strengthening group and 21 to the control group. Two participants withdrew from the strengthening group before the intervention, one due to the nature of the fracture and the other due to patient convenience, and were thus excluded from the analysis. One participant in the strengthening group and one in the control group dropped out during the follow-up period because of work demand and pneumonia, respectively. In total, 40 participants (20 in each group) were analyzed (Fig. 5). Two participants in the strengthening group and two in the control group regularly exercised in their daily lives, but none performed specific exercises for low back pain. Table 1 shows the baseline characteristics of the patients who were enrolled in the trial. No significant differences in age, sex, or other variables were observed between the two groups using the chi-squared test and the Mann–Whitney U test (Table 1). Notably, no adverse events related to the exercise were reported.

**Table 1:** Baseline characteristics of participants in both groups.

Baseline characteristics	Strengthening group n = 20	Control group n = 20	*P* value
Age	73.0 (68.5–77.0)	77.0 (70.5–79.3)	0.198
Sex (male/female)	5/15	5/15	–
BMI (kg/m^2^)	23.6 (21.9–25.6)	24.3 (21.1–26.0)	0.685
Abdominal trunk muscle strength (kPa)	4.8 (3.7–6.6)	4.3 (2.7–5.5)	0.394
Low back pain NRS	7.0 (6.8–8.0)	7.0 (5.0–7.3)	0.201
RDQ	9.0 (7.0–11.0)	9.5 (8.0–12.3)	0.663
FFD (cm)	0.0 (− 5.3 to 1.3)	− 3.0 (− 10.0 to 0.0)	0.262
TUG test (s)	6.3 (6.1–6.8)	7.3 (6.1–7.9)	0.181
OLS test (s)	37.6 (16.4–60.0)	16.4 (5.3–60.0)	0.165
**SF-36**			
Physical functioning	32.6 (21.7–43.4)	27.2 (21.7–36.2)	0.384
Role physical	29.1 (22.5–35.8)	29.1 (24.2–36.6)	0.989
Bodily pain	35.4 (29.9–35.5)	31.4 (29.9–35.4)	0.202
General health perceptions	46.9 (39.8–52.2)	44.2 (37.8–47.5)	0.220
Vitality	45.0 (39.4–46.6)	43.4 (36.2–46.6)	0.732
Social functioning	37.7 (29.6–50.6)	31.2 (31.2–44.1)	0.760
Role emotional	43.6 (31.1–56.1)	43.6 (35.3–56.1)	0.726
Mental health	43.8 (38.4–51.8)	41.1 (35.7–47.1)	0.349

Data are shown as the median and interquartile range or as the number as appropriate.

*BMI* body mass index, *NRS* numerical rating scale, *RDQ* The Roland Morris Disability Questionnaire, *FFD* Finger-floor distance, *TUG* Timed up and go, *OLS* one-leg standing, *SF-36* Short-Form 36-Item.

### Primary outcomes

**Figure 6:**
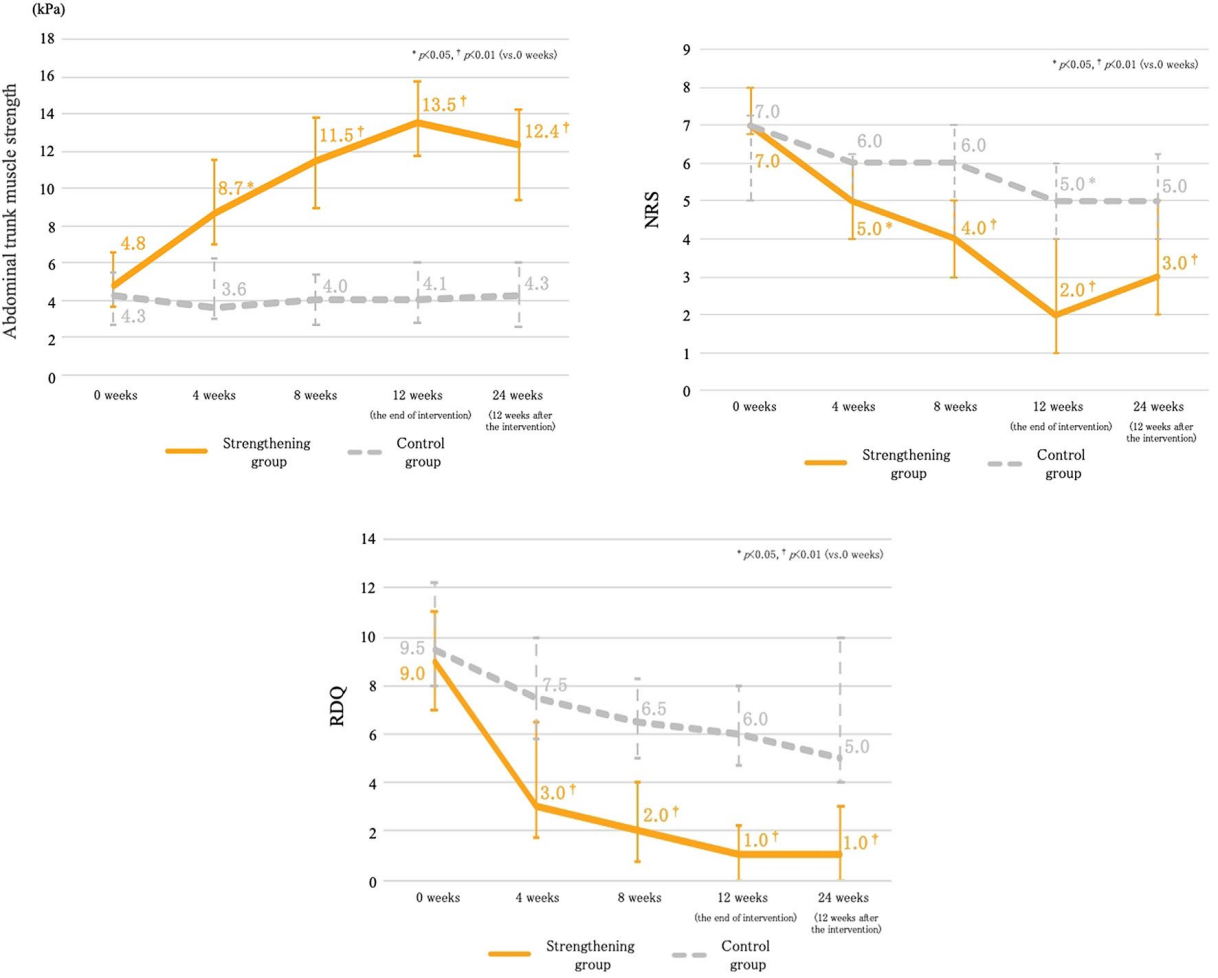
(**a**) The graph illustrates the change in abdominal trunk muscle strength during the trial. Median abdominal trunk muscle strength measured at 4, 8, 12, and 24 weeks were significantly higher than those measured at baseline in the strengthening group; no significant change was observed in the control group. (**b**) The graph illustrates the change in NRS of LBP during the trial. Median NRS measured at 4, 8, 12, and 24 weeks were significantly lower than those measured at baseline in the strengthening group. Those measured at 12 weeks were significantly lower than baseline in the control group. (**c**) The graph illustrates the change in RDQ during the trial. Median RDQ measured at 4, 8, 12, and 24 weeks were significantly lower than those measured at baseline in the strengthening group.

Figure 6 illustrates the changes in the abdominal trunk muscle strength, NRS of LBP, and RDQ during the trial. The median abdominal trunk muscle strength measured at 4, 8, 12, and 24 weeks were significantly higher than those measured at baseline in the strengthening group; no significant change was observed in the control group. The median NRS and RDQ measured at 4, 8, 12, and 24 weeks were significantly lower than those measured at baseline in the strengthening group. The median NRS measured at 12 weeks was significantly lower than that measured at baseline in the control group. No significant change in RDQ was observed in the control group. These results demonstrated that the abdominal trunk muscle strength and LBP improved after 4 weeks of strengthening exercise using the device, and these improvements were maintained at 12 weeks after the intervention. There was no significant difference in improvement in NRS of CLBP, RDQ, or abdominal trunk muscle strength between those who exercised in the strengthening group and those who did not.

### Secondary outcomes

Table 2 summarizes the outcome measures in each group at baseline and 12 weeks. Significant improvement in FFD and TUG at 12 weeks was observed compared to the baseline values in both groups. OLS at 12 weeks was significantly longer than that at baseline in the strengthening group. All subscales of the SF-36 improved in the strengthening group; however, only five of the eight subscales improved in the control group.

**Table 2:** Outcomes at baseline and after the 12-week intervention of both groups.

Outcomes	Strengthening group n = 20			Control group n = 20		
	0-week (Baseline)	12-week (the end of intervention)	*P* value	0-week (Baseline)	12-week (the end of intervention)	*P* value
Abdominal trunk muscle strength (kPa)	4.8 (3.7–6.6)	13.5 (11.8–15.7)	< 0.01	4.3 (2.7–5.5)	4.1 (2.8–6.1)	0.99
Low back pain NRS	7.0 (6.8–8.0)	2.0 (1.0–4.0)	< 0.01	7.0 (5.0–7.3)	5.0 (4.0–6.0)	0.049
RDQ	9.0 (7.0–11.0)	1.0 (0.0–2.3)	< 0.01	9.5 (8.0–12.3)	6.0 (4.8–8.0)	0.057
FFD (cm)	0.0 (− 5.3–1.3)	6.0 (0.4–15.0)	< 0.01	− 3.0 (− 10.0 to 0.0)	1.0 (− 7.5 to 5.5)	< 0.01
TUG test (s)	6.3 (6.1–6.8)	5.7 (5.0–6.2)	< 0.01	7.3 (6.1–7.9)	7.0 (5.9–7.9)	0.011
OLS test (s)	37.6 (16.4–60.0)	52.7 (27.6–60.0)	< 0.01	16.4 (5.3–59.6)	18.7 (9.0–54.8)	0.099
**SF-36**						
Physical functioning	32.6 (21.7–43.4)	48.8 (38.9–51.5)	< 0.01	27.2 (21.7–36.2)	34.4 (24.5–47.0)	0.013
Role physical	29.1 (22.5–35.8)	49.1 (39.9–53.2)	< 0.01	29.1 (24.2–36.6)	35.8 (28.3–40.8)	< 0.01
Bodily pain	35.4 (29.9–35.5)	47.4 (44.7–54.6)	< 0.01	31.4 (29.9–35.4)	35.4 (35.4–39.9)	< 0.01
General health perceptions	46.9 (39.8–52.2)	54.8 (51.5–60.2)	< 0.01	44.2 (37.8–47.5)	46.3 (41.5–50.2)	0.234
Vitality	45.0 (39.4–46.6)	56.3 (52.2–60.3)	< 0.01	43.4 (36.2–46.6)	45.0 (43.4–47.4)	0.093
Social functioning	37.7 (29.6–50.6)	50.6 (44.1–57.0)	< 0.01	31.2 (31.2–44.1)	44.1 (31.2–50.6)	0.092
Role emotional	43.6 (31.1–56.1)	56.1 (46.7–56.1)	< 0.01	43.6 (35.3–56.1)	45.7 (43.6–56.1)	0.033
Mental health	43.8 (38.4–51.8)	54.5 (45.8–62.6)	< 0.01	41.1 (35.7–47.1)	46.5 (43.1–51.8)	< 0.01

Data are presented as the median and interquartile range.

*NRS* numerical rating scale, *RDQ* The Roland Morris Disability Questionnaire, *FFD* Finger-floor distance, *TUG* Timed up and go, *OLS* one-leg standing, *SF-36* Short-Form 36-Item.

### Comparison between the two groups

Table 3 shows the improvement in the primary and secondary outcomes between the baseline and the end of the intervention (at 12 weeks in each group). The median of improvement in the abdominal trunk muscle strength, NRS of LBP, RDQ, and TUG in the strengthening group were significantly better than those in the control group. Improvement in six of the eight subscales of the SF-36 was better in the strengthening group than in the control group.

**Table 3:** Comparison of the improvement after 12-week intervention between each group.

Outcomes	Strengthening group n = 20	Control group n = 20	*P* value
Δ Abdominal trunk muscle strength (kPa)	9.4 (7.1–11.0)	0.3 (− 0.2 to 0.8)	< 0.01
Δ Low back pain NRS	4.0 (3.0–6.0)	1.0 (1.0–2.0)	< 0.01
Δ RDQ	7.5 (6.0–9.0)	3.0 (0.8–5.0)	< 0.01
Δ FFD (cm)	7.0 (3.4–10.3)	4.5 (2.8–5.0)	0.096
Δ TUG test (s)	0.8 (0.5–1.1)	0.2 (0.0–0.5)	< 0.01
Δ OLS test (s)	1.3 (0.0–16.0)	0.9(− 0.1 to 3.5)	0.799
**SF-36**			
Δ Physical functioning	7.2 (3.6–16.2)	3.6 (0.0–7.2)	0.04
Δ Role physical	18.3 (8.3–23.3)	3.3 (0.0–7.5)	< 0.01
Δ Bodily pain	11.8 (4.9–22.5)	4.5 (0.0–8.5)	< 0.01
Δ General health perceptions	5.3 (2.7–12.1)	0.0 (− 0.3 to 2.9)	< 0.01
Δ vitality	14.5 (6.4–19.3)	0.0 (− 0.8 to 7.2)	< 0.01
Δ Social functioning	9.7 (0.0–20.9)	0.0 (0.0–6.4)	< 0.01
Δ Role emotional	4.2 (0.0–13.5)	0.0 (0.0–5.2)	0.057
Δ Mental health	6.7 (0.0–16.8)	5.4 (0.0–8.7)	0.287

Data are shown as the median and interquartile range.

*NRS* numerical rating scale, *RDQ* The Roland Morris Disability Questionnaire, *FFD* Finger-floor distance, *TUG* Timed up and go, *OLS* one-leg standing, *SF-36* Short-Form 36-Item.

No significant difference in the median total number of days on which the participants performed exercise sessions and home stretching exercises between the groups was observed (82.0, 81.0–84.0 days out of 84 days in the strengthening group; 84.0, 78.8–84.0 days in the control group). This result indicated that both groups demonstrated optimal adherence to the respective exercise programs and similar interventions except for the strengthening exercise.

## Discussion

Strengthening exercise using the device is similar to abdominal bracing under pressure. Abdominal bracing is a type of lumbar stabilization exercise that aims to contract the entire abdominal core muscles to provide great spine stability^22,39,40^. In abdominal bracing, co-contraction of the deep core muscles increases the intra-abdominal pressure from the hoop created via the thoracolumbar fascia, which in turn adds stiffness to the spine^41^. All trunk muscles were reported to play an important role in achieving spine stability and must work harmoniously to reach this goal^40,42^. Abdominal contraction (bracing) was also shown to be one of the most effective exercise techniques for trunk stabilization training^39^.

Strengthening exercise with this device could activate all core muscles (described as the “muscular box”,) which consist of the anterolateral aspect, the roof, and the floor of the abdominal trunk muscles^20^. Therefore, strengthening the muscular box of the core using the device could increase the stability of the lumbar spine and improve LBP. Moreover, LBP improvement and increased spine stability with strengthening exercise might improve dynamic balance, functional mobility, and QOL.

In this study, the improvement in LBP and abdominal trunk muscle strength by strengthening exercise using the device was maintained at 12 weeks after the intervention. Thus, the strengthening exercise with the device may also be effective in preventing LBP recurrence.

In this clinical trial, the strengthening exercise using the device was performed in a sitting position without painful movement of the lower back, and the exercise did not induce pain in the trunk or extremities in any patient, including the elderly. Additionally, the device could be a viable option for measuring core muscle strength, as the measurement is easy with the device and it has an excellent intra-rater and inter-rater reliabilities^20^. Furthermore, muscle activation during exercise could be monitored in real-time in waveform and numerical form with the device, which, in turn, could facilitate patients’ performance of appropriate muscle contractions.

As the exercise using the device could be easily performed even among the elderly, the exercise could be performed at home hereafter. All these advantages can enhance patient adherence and improve the effectiveness of the exercise, and consequently, the economic burden related to LBP treatment can be reduced.

Various lumbar stabilization exercises have been reported to be effective in reducing LBP, including plank, bridging, or machine exercise, but most studies generally have younger participants compared with our study^43–47^. Many elderly patients with CLBP cannot perform the same intensity of exercise as younger people due to pain and deteriorated physical function^48,49^. Exercise using our device is easy to perform, even for the elderly, and resulted in improvements in LBP even considering the advanced age of our participants and that they do not exercise in their daily lives. Therefore, exercise using our device is more suitable for elderly patients than other exercises.

A statistically significant difference does not necessarily mean that the difference is clinically substantial. MCID is defined as the smallest change that is important to patients^50^. If improvements in patients' score with treatment do not exceed the MCID thresholds, the treatment is considered to be insufficient for the condition^51^. MCID of two points on the NRS of LBP and MCID of four points on the RDQ could accurately distinguish patients who have demonstrated a clinically meaningful change^25,28,52–55^.

For NRS, 95% of the participants in the strengthening group and 45% of the participants in the control group achieved the MCID threshold of two points. For RDQ, 85% of the participants in the strengthening group and 45% of the participants in the control group achieved MCID. Therefore, LBP improvement with the strengthening exercise using the device could be considered clinically significant.

Effect size (Cohen's d) is a quantitative measure of the magnitude of experimenter effect independent of sample size. The larger the effect size, the stronger the relationship between two variables. Cohen et al. suggested that values of d of 0.2, 0.5, 0.8, 1.20, and 2.0 could be considered small, medium, large, very large, and huge effect sizes, respectively^56,57^. In this study, the effect size of the strengthening exercise using the device in improving NRS of LBP was 1.98, indicating that the intervention was highly effective.

In this trial, the 12-week exercise using the device did not result in any adverse events. This result showed that elderly and young patients could safely use this device. However, the device induces increased intra-abdominal pressure. Thus, the use of the device should be prohibited in patients with a history of abdominal surgery and abdominal hernia. Moreover, great care should be taken for patients with hypertension or cardiovascular, respiratory, cerebrovascular, gynecological, or other medical diseases.

In this study, we measured abdominal trunk muscle strength using our new device only. Other conventional measurement methods (e.g., dynamometers) might exacerbate LBP in patients with CLBP. Future studies to compare the measurements using our new device and other conventional methods are needed to further understand abdominal trunk muscle strength.

The main limitation of this study was the absence of randomization. We opted to employ a non-randomized design because it was more feasible than a randomized design from the institutional circumstances. However, it should be noted that none of the baseline variables differed significantly between the groups.

Another limitation was the difference in the number of supervised sessions between the groups. To minimize this bias, we encouraged all participants to perform home stretching exercises every day. Although the number of hospital visits differed, both groups demonstrated good adherence to the exercise program; notably, no significant difference in adherence was observed between the groups.

## Conclusion

The strengthening exercise using the device with easy stretching was effective in improving the NRS of CLBP, abdominal trunk muscle strength, physical function, and QOL. This new exercise program using the novel device is effective in the treatment for CLBP.

## Data Availability

The study data and details of materials used may be made available upon reasonable request by sending an e-mail to the first author.
